# Linkages between atmospheric blocking, sea ice export through Fram Strait and the Atlantic Meridional Overturning Circulation

**DOI:** 10.1038/srep32881

**Published:** 2016-09-13

**Authors:** M. Ionita, P. Scholz, G. Lohmann, M. Dima, M. Prange

**Affiliations:** 1 Alfred Wegener Institute Helmholtz Center for Polar and Marine Research, Bremerhaven, Germany; 2 MARUM – Center for Marine Environmental Sciences, University of Bremen, Bremen, Germany; 3 Bucharest University, Faculty of Physics, Bucharest, Romania

## Abstract

As a key persistent component of the atmospheric dynamics, the North Atlantic blocking activity has been linked to extreme climatic phenomena in the European sector. It has also been linked to Atlantic multidecadal ocean variability, but its potential links to rapid oceanic changes have not been investigated. Using a global ocean-sea ice model forced with atmospheric reanalysis data, here it is shown that the 1962–1966 period of enhanced blocking activity over Greenland resulted in anomalous sea ice accumulation in the Arctic and ended with a sea ice flush from the Arctic into the North Atlantic Ocean through Fram Strait. This event induced a significant decrease of Labrador Sea water surface salinity and an abrupt weakening of the Atlantic Meridional Overturning Circulation (AMOC) during the 1970s. These results have implications for the prediction of rapid AMOC changes and indicate that an important part of the atmosphere-ocean dynamics at mid- and high latitudes requires a proper representation of the Fram Strait sea ice transport and of the synoptic scale variability such as atmospheric blocking, which is a challenge for current coupled climate models.

Sea ice is an important component of the Arctic climate system, affecting heat, freshwater and momentum fluxes between the ocean and the atmosphere. Huge amounts of sea ice and freshwater are transported through Fram Strait, which connects the Arctic Ocean and the northern North Atlantic sector, and influence water densities in these regions. Variations in Fram Strait Sea Ice Export (FSSIE) have been associated with the Great Salinity Anomaly (GSA) observed in the late 1960s to early 1970s in the North Atlantic^1^,^2^,^3^.

The atmospheric circulation over the Arctic plays a key role in the evolution of sea ice growth, movement and melting. Half of the variance in the summer sea ice extent over the past three decades has been influenced by lower atmospheric winds^4^. Anomalous sea ice motion through Fram Strait is also largely driven by atmospheric forcing, linked to wind and sea level pressure (SLP) anomalies^5^,^6^,^7^. Over the 1979–1997 period, FSSIE was strongly related to the North Atlantic Oscillation (NAO)^8^,^9^, but for previous time intervals the influence of NAO on the sea ice export had been almost insignificant^8^,^10^. NAO is seen as a natural mode of variability intrinsic to the Northern Hemisphere climate. However, NAO can react to different external forcing (e.g. volcanic and/or solar activity, greenhouse gases)^11^. Recent studies have shown that the origin of NAO resides in the presence of Rossby Wave Breaking (RWB) events. At the same time, RWBs have been associated with the occurrence of atmospheric blocking events^12^. Moreover, it has been shown that the polarity of NAO is associated with Greenland blocking episodes: the negative phase of NAO is strongly influenced by the presence of blocking activity over Greenland, while the NAO positive phase is associated with reduced Greenland blocking activity^13^,^14^,^15^. Other large-scale atmospheric patterns that were found to play a role in FSSIE variability are an east-west sea level pressure dipole pattern with one center over the Kara/Laptev Seas and another center over the Canadian Arctic Archipelago^16^,^17^ and particular cyclone trajectories^18^. Based on atmospheric pressure data, Walsh and Chapmann^19^ identified a relation between the GSA in the 1970s and strong FSSIE, in conjunction with an anomalous surface pressure pattern over the Arctic and East of Greenland, of unknown origin.

On interdecadal timescales, NAO also plays a role in modulating the AMOC variability^20^,^21^, while on multidecadal timescales AMOC is closely related to the Atlantic Multidecadal Oscillation (AMO) (which on multidecadal time scales is also related to NAO)^22^,^23^,^24^. To complete the full picture of the NAO, AMO and AMOC interplay as well as the corresponding ocean-atmosphere interactions on different timescales, the use of coupled climate models would be desirable. However, the main issue with the use of coupled models to study the relationship between AMOC and atmospheric forcing (e.g. NAO and/or atmospheric blocking) is the marked biases that exist in the representation of NAO and atmospheric blocking in climate models^25^,^26^,^27^. Recent studies^25^,^26^,^27^ have shown that even the new generation of climate models (Coupled Model Intercomparison Project Phase 5 – CMIP5)^28^ tend to underestimate the blocking activity over Europe and Greenland and the physical processes connected to the NAO. Here, we investigate the potential driving role of a “persistent” atmospheric blocking activity for FSSIE as well as the associated consequences for the North Atlantic freshwater budget and ocean circulation in this sector in an uncoupled ocean model.

The study relies on the Finite-Element Sea-Ice Ocean Model (FESOM)^29^ with a setup configuration that includes an enhanced resolution in the northern hemisphere deep-water formation areas^30^,^31^, which is crucial for a realistic implementation of the deep ocean ventilation (see Supplementary file). The model is forced with atmospheric data from the Coordinated Ocean Ice Reference Experiment version 2 (COREv2)^32^, which allows a realistic simulation of sea ice transport variations through Fram Strait, including the outstanding high sea ice transport around 1967–1969, followed by smaller events around 1975, 1981, 1989 and 1993–1995 (Fig. 1). The simulated December-January-February (DJF) averaged FSSIE variability matches the observed time series of Schmith and Hansen^33^ with a correlation coefficient of 0.61 (0.001 significance level). The correlation between the modeled FSSIE and the observed FSSIE is not significant for other seasons (e.g. spring and autumn, Table S1) or is smaller compared to the winter season (e.g. summer and annual, Table S1). As such, in this study we base our analysis on the winter (DJF) modeled FSSIE.

## Atmospheric blocking and sea ice export

Atmospheric blocking is a large-scale mid-latitude atmospheric phenomenon mostly associated with persistent quasi-stationary synoptic-scale high-pressure systems. It may cause large-scale circulation anomalies exerting a strong impact on weather patterns and is therefore often associated with significant climate anomalies^34^,^35^,^36^. In order to investigate the relationship between atmospheric blocking activity and modeled DJF FSSIE variations, we evaluate the North Atlantic sector 2D blocking composite maps for years when the time series of FSSIE was **higher** (*lower*) than **0.75** (*−0.75*) standard deviations. To emphasize the variation in the relationships between the two variables, the simultaneous and lagged relationship, with a lag of up to 5 years (FSSIE lags) when the FSSIE was higher than 0.75 standard deviations, is analyzed.

**Figure 1:**
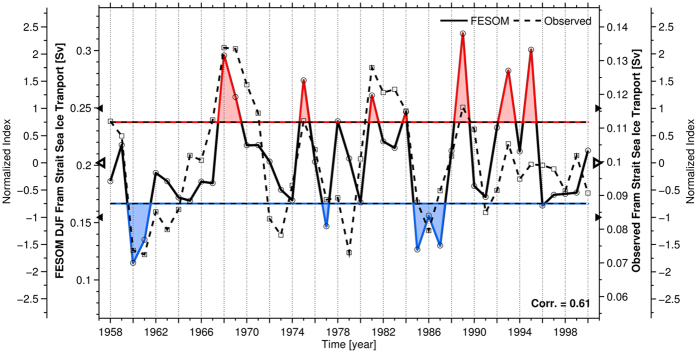
Absolute and normalized, modeled winter (solid) and observed (dashed^33^) Fram Strait sea-ice export time-series for the interval 1958 to 2000. Periods when the modeled Fram Strait sea-ice export was above and below 0.75 standard deviation are indicated by red and blue shadings, respectively. Empty (filled) triangles indicate the values for the mean (standard deviation) of the modeled and observed Fram Strait sea-ice export time-series. Figure 1 has been produced with MATLAB software – version 2014b (http://de.mathworks.com/products/new_products/release2014b.html).

To analyze the influence of atmospheric blocking activity over Greenland and Northern Europe on sea ice advection in the Arctic Ocean, we calculated the stream function Ψ of the divergence-free part of the sea ice thickness vector field **v** = (h·u, h·v), where h is the sea ice thickness and u, v denote the ice velocity components, multiplied with the sea ice concentration c, by solving the Poisson equation ΔΨ = −curl(c·**v**). This stream function accounts only for the horizontal advective sea ice transport, but not for contributions from source terms like freezing and melting. Advective sea ice transport occurs along the lines of constant stream function values. Positive (negative) stream function values characterize a clockwise (counter clockwise) circulation. We refer to this stream function as the advective sea ice stream function. The lagged (high) and in phase (high and low) composite maps for the blocking frequency, the Arctic sea ice thickness and the advective sea ice stream function were computed based on the simulated FSSIE time series (Fig. 1).

**Figure 2:**
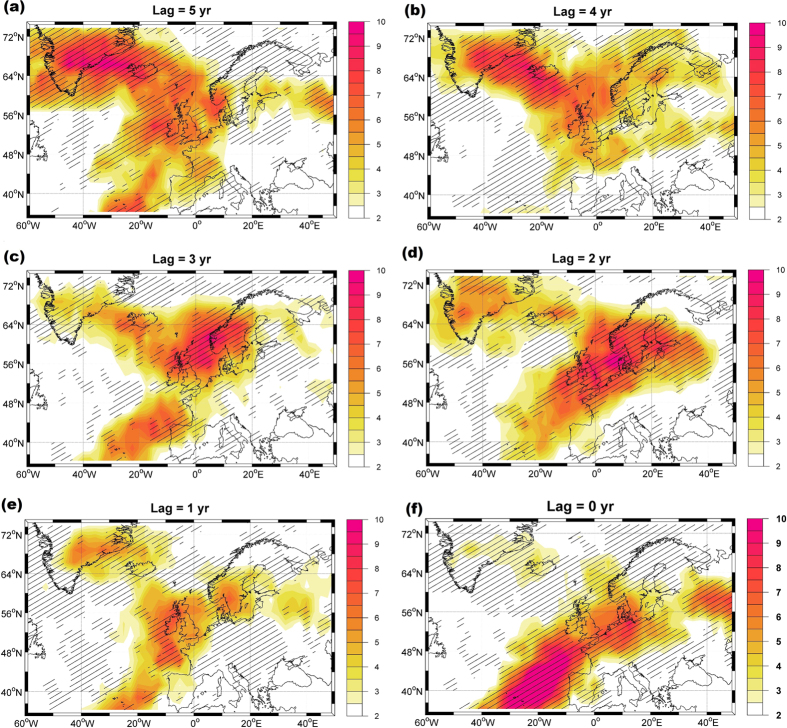
(**a–f**) 2D atmospheric blocking frequency high composite maps for winter (DJF),with respect to the modeled DJF Fram Strait sea-ice export time-series above 0.75 standard deviation for different time lags between five (**a**) and zero (**f**) years (Fram Strait sea-ice export time-series lags). The hatching highlights significant anomalies at a confidence level of 95%. Figure 2 has been produced with MATLAB software – version 2014b (http://de.mathworks.com/products/new_products/release2014b.html).

Starting five years before high DJF FSSIE, a center of enhanced blocking activity over Greenland, coupled to a center of weaker blocking activity over Northern Europe, is observed (Fig. 2a). The blocking activity over Greenland vanishes with decreasing lag, while the center of the blocking activity over Northern Europe remains and subsequently couples to a center of enhanced blocking activity over the North Atlantic (Fig. 2b–e). At zero lag (in phase relationship), when there is high FSSIE, the center of blocking activity is entirely shifted to Northern Europe and the North Atlantic with no blocking over Greenland (Fig. 2f). The in- phase high (>0.75 standard deviation) and low (<−0.75 standard deviation) composite maps between the atmospheric blocking activity and the modeled FSSIE (Figure S6a,b) show that low FSSIE is associated with a coupled Greenland – Northern Europe blocking activity, while high FSSIE is linked to a coupled Northern Europe – North Atlantic blocking activity. The former pattern leads to a reduced (“blocked”) sea ice transport over the Greenland Sea and supports the accumulation of Arctic sea ice (Figure S6c), while the latter pattern favors enhanced sea ice advection and the outflow of Arctic sea ice through Fram Strait (Figure S6d). The same results are obtained when using the reconstructed FSSIE time series^33^ for the composite map analysis over the 1948–2000 period (Figure S8).

**Figure 3:**
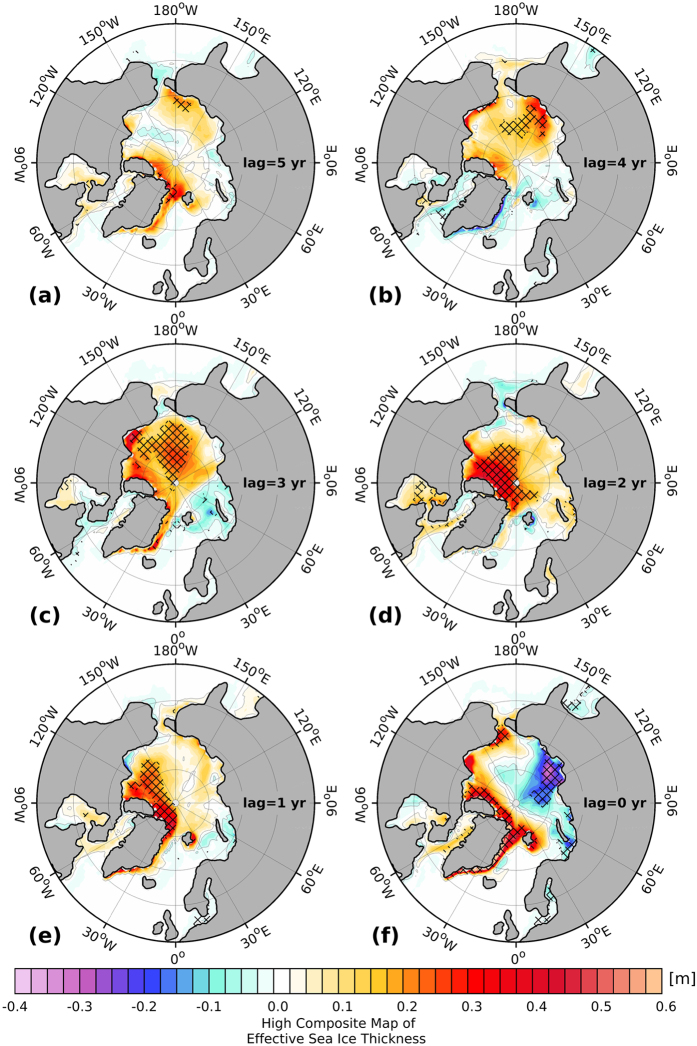
(**a–f**) Arctic sea ice thickness anomaly high composite maps for winter (DJF), with respect to the modeled DJF Fram Strait sea-ice export time-series above 0.75 standard deviation for different time lags between five (**a**) and zero (**f**) years (Fram Strait sea-ice export time-series lags). The hatching highlights significant anomalies at a confidence level of 95%. Figure 3 has been produced with MATLAB software – version 2014b (http://de.mathworks.com/products/new_products/release2014b.html).

The modeled sea ice thickness (Fig. 3) indicates that five years before high FSSIE (Fig. 3a) positive sea ice thickness anomalies are present especially in the East Siberian Sea and in the Laptev Sea, as well as around the northern and eastern coasts of Greenland. Until two years before high FSSIE (Fig. 3d), a further accumulation of sea ice thickness in the central Arctic is detected. Thereafter, the maximum in the accumulated sea ice relocates towards the Beaufort Sea, north of Greenland, and the Canadian Arctic Archipelago (CAA). At lag 0, the modeled sea ice thickness (Fig. 3f) features positive anomalies in the Fram Strait and at the western Arctic Coast as well as strong negative anomalies in the Laptev Sea. The in-phase low composite map between the modeled sea ice thickness and the FSSIE (Figure S7a) reveals that during these phases the sea ice accumulates along the eastern Arctic coast, while negative anomalies are found north of Greenland and in Fram Strait.

**Figure 4:**
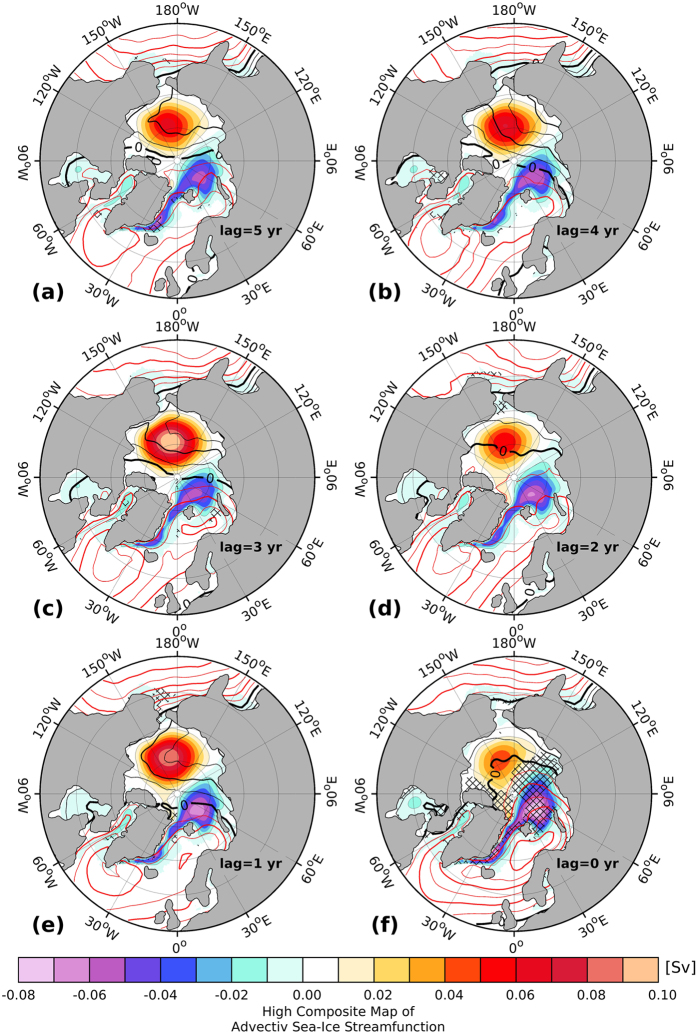
(**a–f**) Advective sea ice streamfunction (shaded) high composite maps for winter (DJF), with respect to the modeled DJF Fram Strait sea-ice export time-series above 0.75 standard deviation for different time lags between five (**a**) and zero (**f**) years (Fram Strait sea-ice export time-series lags). Positive and negative values indicate a clockwise and counterclockwise directed circulation, respectively. Contour lines mark the high composite map of the SLP anomaly (units: hPa), where black lines indicate positive and red lines negative anomalies with an interval of 1.5 hPa. Figure 4 has been produced with MATLAB software – version 2014b (http://de.mathworks.com/products/new_products/release2014b.html).

The advective sea ice stream function features a general decrease (increase) in the strength of the Beaufort Gyre (Transpolar Drift) (Fig. 4a–f) for high FSSIE years. The Arctic high pressure anomaly (black contour) indicates a general weakening, while the Icelandic Low pressure anomaly (red contour) intensifies and reaches its maximum extent in the Laptev Sea. Nevertheless, one and three years before high FSSIE, there is also some partial regeneration in the strength of the Beaufort Gyre and the Arctic high pressure anomaly as well as a partial reduction in the strength of Transpolar Drift and Icelandic Low pressure system. The most obvious characteristic of the lagged composite is the direction of the streamlines in the central Arctic. Between one and up to five years before high FSSIE, the direction of the streamlines in the central Arctic tends to be directed to the northern part of Greenland and the CAA. The direct comparison of the in-phase high and low composite maps between the advective sea ice stream function and the FSSIE (Figure S6c,d) shows, for the low composite map (Figure S6c), a strong Beaufort Gyre and Arctic high pressure anomaly, as well as a weak Transpolar Drift and Icelandic Low pressure system, when compared to the high composite map (Figure S6d). The direction of the streamlines in the central Arctic, for the high composite map, points into Fram Strait, while for the low composite map they are directed to the north of Greenland and the CAA.

In summary, reduced FSSIE is associated with a center of enhanced blocking activity over Greenland, coupled to a persistent center of enhanced blocking activity over Northern Europe (Fig. 2a). This kind of structure leads to a reduced (‘blocked’) meridional air mass exchange over the Greenland Sea and to a reduction of the northerly surface wind stress (Figure S7c), which in turn diminishes the sea ice transport through Fram Strait and leads to an accumulation of sea ice in the Arctic Ocean. Enhanced blocking activity over Greenland reduces the sea ice transport towards Fram Strait (Figure S6c), so that negative sea ice thickness anomalies are observed in this area, whereas positive, but insignificant, anomalies are found in the Laptev, East Siberian and Chukchi Seas (Figure S7a). These conditions are accompanied by minimum sea ice thickness, northward wind stress anomalies and reduced horizontal (barotropic) stream function along the East Greenland current (Figure S7a,c,e). This configuration implies a reduction of the freshwater export from the Arctic, which can accumulate in the Arctic basin. In addition, the weakened oceanic circulation in the Nordic Seas, as inferred from the horizontal barotropic stream function (Figure S7e), points to a reduced inflow of warm Atlantic Water (i.e. heat) into Fram Strait and Barents Sea, which can have an additional impact on Arctic sea ice extent and volume^37^,^38^.

Enhanced FSSIE is associated with strongly reduced blocking activity over Greenland and enhanced blocking activity over the eastern North Atlantic (Fig. 2f). This pattern enables a meridional air-mass exchange over the Greenland Sea and favors northeasterly wind stress promoting sea ice advection from the Arctic Ocean towards the North Atlantic (Figure S7b). Reduced blocking activity over Greenland enhances the sea ice transport towards Fram Strait (Figure S6d), so that sea ice accumulates in this area (Figure S6d). These conditions are accompanied by maximum sea ice thickness, southward wind stress anomalies and enhanced horizontal barotropic stream function along the East Greenland Current (Figure S7b,d,f). Such a configuration implies a freshwater flush out of the Arctic (Figure S6d). In addition, the accelerated oceanic circulation in the Nordic Seas (Figure S7f) points to an increased inflow of warm Atlantic Water into the Arctic Ocean with potential impact on Arctic sea ice extent and volume.

## Atmospheric blocking and the 1960s Great Salinity Anomaly

The second half of the 20^th^ century witnessed a series of decadal-scale anomalies of salinity, temperature and sea ice cover in the northern North Atlantic^1^,^2^,^39^. The most pronounced one, the GSA which occurred in the late 1960s to the early 1970s, was largely caused by intense sea ice export from the Arctic Ocean through the Fram Strait^1^,^34^,^40^. During GSA events, low-salinity surface water propagates into the Labrador Sea and reduces the deep water formation there which leads, as a consequence, to a weakening of the AMOC and the deep western boundary current^41^.

Although the causes of 1970’s GSA event are partially known^39^, no study has made a direct link between the GSA, the AMOC, FSSIE and their relationship with persistent atmospheric blocking. From an atmospheric point of view, the 1970’s GSA event was related to the presence of a persistent negative NAO, but not all GSA events occurred during a negative phase of the NAO^1^. Since NAO is strongly dependent on the frequency of Greenland blocking^15^ and RWB events^13^, one needs to consider the atmospheric drivers of the GSA events not just from an NAO point of view. As such, the identified link between atmospheric blocking and FSSIE complements the causal chain between changes in AMOC, GSA events, FSSIE and atmospheric forcing.

**Figure 5:**
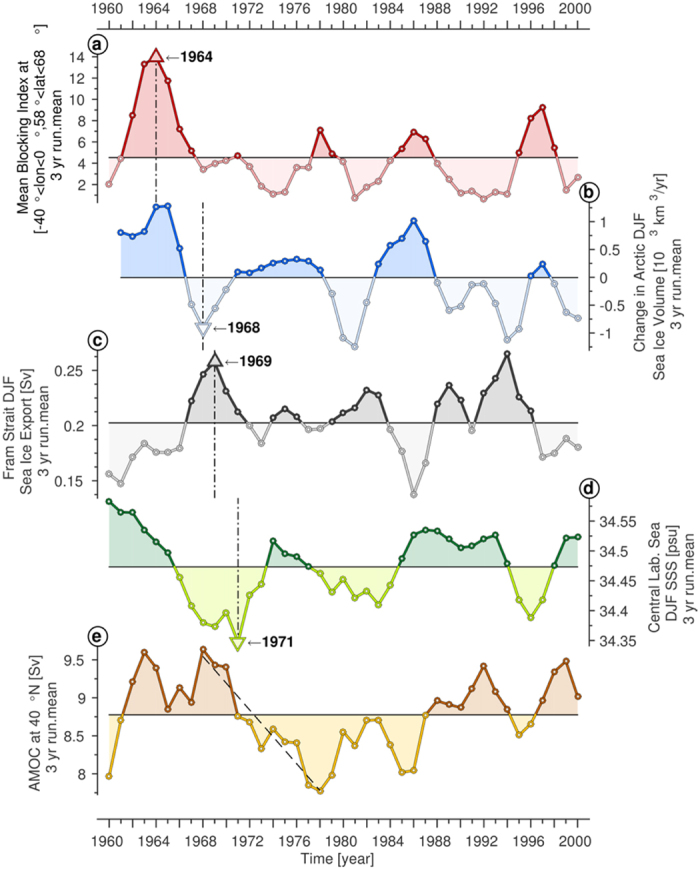
The 3 year-running mean filtered time-series (from top to bottom) of the mean blocking index at (−40° < lon < 0°, 58° < lat < 68°) (**a**), change in modeled DJF Arctic sea-ice volume (**b**), modeled DJF Fram Strait sea ice export (**c**), modeled DJF sea surface salinity in the central Labrador Sea (**d**) and modeled maximum annual AMOC at 40°N (**e**). Figure 5 has been produced with MATLAB software – version 2014b (http://de.mathworks.com/products/new_products/release2014b.html).

**Figure 6:**
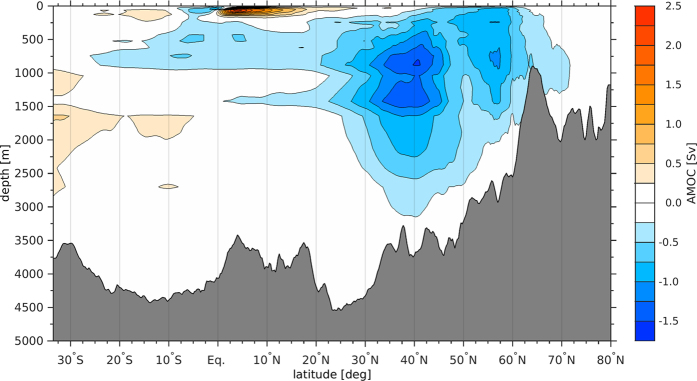
Anomaly of the mean Atlantic Meridional Overturning Circulation (AMOC) streamfunction: 1972–1986 minus 1962–1970. Figure 6 has been produced with MATLAB software – version 2014b (http://de.mathworks.com/products/new_products/release2014b.html).

The mean blocking frequency, averaged over the region −40°E–0°E, 48°N–58°N for the 1960–2001 period (Fig. 5a), shows that the strongest Greenland blocking event within the last five decades occurred between 1962 and 1966 (Fig. 5a and Figure S4a). The prominent blocking configuration over Greenland (Figure S5) is concurrent with a positive change of the mean DJF Arctic sea ice volume in the model (Fig. 5b) indicating an accumulation of Arctic sea ice. Upon the weakening of the Greenland blocking after 1966, the accumulated sea ice flushes out of the Arctic through FSSIE and the Arctic sea ice volume decreases (Fig. 5b,c). The DJF FSSIE reaches its maximum around 1968–1969, which coincides with a strong reduction of the Greenland blocking (Fig. 5a and Figure S4b) and one of the lowest values in the change of the Arctic sea ice volume. The high values of FSSIE and the resulting sea ice melting led to an additional freshwater input into the East Greenland Current. To exclude the possibility of a significant oceanic fresh water input into the East Greenland Current from the Arctic, we calculated the oceanic mean liquid fresh water transport out of the Arctic through Fram Strait, at a reference salinity of 35 psu. This revealed to be 2.5 times smaller than the contributions from the FSSIE. The resulting melting of the increased sea ice export through Fram Strait leads to a freshwater anomaly that travels within 2 to 3 years towards the central Labrador Sea via the East and West Greenland Currents as well as the Labrador Current. Consequently, between 1968 and 1971 the freshwater anomaly reaches the central Labrador Sea and causes the strongest negative surface salinity anomaly of the last five decades (Fig. 5d, Figure S9b). The freshening of the Labrador Sea surface layers causes a negative haline forcing and hence a decrease in the deep ventilation of the central Labrador Sea with cold and fresh water masses from the surface. This in turn leads to an anomalous warming and an increase in salinity in the intermediate and deeper layers of the central Labrador Sea (Figure S9). Commencing with the arrival of the GSA in the Labrador Sea, a shift in the strength of the modeled maximum AMOC at 40°N from a stronger to a weaker state is observed (Figs 5e and 6).

## Concluding remarks

Using a stand-alone global ocean-sea ice model, with an increased resolution around Greenland and in the deep water formation areas, here it is shown that an atmospheric blocking configuration, extending from Greenland to the northern part of Europe, modulates the sea ice accumulation in the Arctic Ocean and the FSSIE. Correspondingly, high blocking activity during the early-mid 1960s generated an anomalously large sea ice accumulation in the Arctic. The sea ice export through Fram Strait caused the 1970’s GSA event, which in turn weakened the Labrador Sea convection and AMOC.

The enhanced blocking activity stretching from Greenland to Western Europe is related to a warmer and more saline subpolar Atlantic Ocean^34^, caused by a reduced meridional exchange of cold air masses from the Arctic and the reduced freshwater input from the accumulation of Arctic sea ice. However, the FSSIE was related to the AMO^42^, which in turn is linked to AMOC changes^22^. Our results indicate that AMOC shifted towards a weaker state in the 1970s (Fig. 6 and Figure S2), as a result of the sea ice flushes from the Arctic after periods of enhanced blocking activity over Greenland, which is consistent with a concurrent jump in the AMO. Therefore, the accumulated Arctic sea ice induced by atmospheric blocking and its flush into the North Atlantic sector appears to be responsible for a rapid AMOC change and an associated climate shift^39^. Consequently, these processes appear as part of an internal mechanism of AMOC shifts, whose abruptness and amplitude are linked to the persistence of the blocking activity. A similar abrupt shift in the AMOC around 1970’s has been found in observational data^39^,^43^. According to Rahmstorf *et al*.^43^, the minimum AMOC strength observed in the late 1970s was never matched in the last millennium, and represents a very exceptional event. The proposed causes for this abrupt shift are diverse: a prolonged freshening trend in the North Atlantic Ocean^44^, increasing river discharge into the Arctic Ocean^45^, the melting of the Greenland Ice Sheet^43^, aerosol forcing^46^ as well as external forcing mechanisms^21^. Nevertheless, none of the aforementioned studies have taken into account the impact of the atmospheric forcing, more specifically atmospheric blocking, on modulating and partially driving this abrupt shift.

Here, we have shown that persistent atmospheric blocking in winter can lead to abrupt shifts in AMOC variability via excessive sea ice export through Fram Strait, which in turn could also affect the climate over Europe. A weaker AMOC is associated, among other things, with a reduced volume of warm water transported from the tropics towards Europe and hence with colder winters and an increased storminess over Europe^47^. According to modeling results^48^, a shutdown or a substantial slowdown of the AMOC will cause a more general increase of severe weather.

The results presented here point to an internal source for abrupt climate changes, with implications for the prediction of AMOC shifts, since winters with enhanced blocking over Greenland tend to persist for years. They also indicate that an important part of the atmosphere-ocean dynamics at mid- and high latitudes requires a proper representation of the Fram Strait sea ice transport and of the synoptic scale variability such as atmospheric blocking, which is a challenge for current coupled climate models^49^,^50^.

## Methods

### Computation of the 2D blocking frequency

As a measure of local blocking frequency, we have used the two-dimensional (2D) blocking index^51^. To compute the 2D atmospheric blocking index, we used the winter daily 500 mb geopotential height (Z500). This data set was extracted from the NCEP/NCAR reanalysis data^52^ for the 1948–2010 period. The 2D blocking index is an extension of the one-dimensional blocking index^53^ to a two-dimensional map of blocking frequencies at every grid point. For each grid-point, the southern gradient (GHGS) and the northern gradient (GHGN) are evaluated as follows:


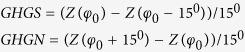


where 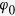 is the latitude of the considered grid point varying from 35°N to 75°N.

For each winter we calculate the ratio between the number of days when a certain grid point was blocked, i.e. the conditions GHGS > 0 and GHGN < (−10m/°.lat) are simultaneously satisfied for at least five consecutive days, and the total number of winter days (90 days). Because we have used Z500 data for 20°N–90°N, the blocking field extends from 35°N to 75°N.

### Composite analysis

To identify the physical mechanism responsible for the connection between the winter sea ice export through Fram Strait and the atmospheric blocking, sea ice thickness, sea ice stream function, and wind-stress, we constructed the composite maps between the normalized time series of sea ice export through Fram Strait for the years when the values of the index were higher than 0.75 std. dev. This threshold was chosen as a compromise between the strength of the climate anomalies associated to sea ice anomalies and the number of maps which satisfy this criterion. Further analysis has shown that the results are not sensitive to the exact threshold value used for our composite analysis (not shown). We have computed composite maps, instead of correlation maps, because the former considers the nonlinearities included in the analyzed data. The significance of the composite maps is based on a standard t-test (confidence level 95%).

## Additional Information

**How to cite this article**: Ionita, M. *et al*. Linkages between atmospheric blocking, sea ice export through Fram Strait and the Atlantic Meridional Overturning Circulation. *Sci. Rep*. **6**, 32881; doi: 10.1038/srep32881 (2016).

## Supplementary Material

Supplementary Information
