# Supplementary material for: Linkages between atmospheric blocking, sea ice export through Fram Strait and the Atlantic Meridional Overturning Circulation
Source: Sci Rep. 2016 Sep 13;6:32881. doi: 10.1038/srep32881 (PMC5020648; doi:10.1038/srep32881)
Supplement: Supplementary Information [file srep32881-s1.pdf]

Supplementary files

Linkages between atmospheric blocking, sea ice export through Fram Strait and the Atlantic Meridional Overturning Circulation

M. Ionita* [1,2], P. Scholz[1,2], G. Lohmann[1,2], M. Dima[1,3] and M. Prange[2]

(1) Alfred Wegener Institute Helmholtz Center for Polar and Marine Research, Bremerhaven, Germany

(2) MARUM – Center for Marine Environmental Sciences, University of Bremen, Bremen, Germany

(3) Bucharest University, Faculty of Physics, Bucharest, Romania

*Corresponding author:

Email: Monica.Ionita@awi.de

Address: Alfred Wegener Institute Helmholtz Centre for Polar and Marine Research
Bussestrasse 24
D-27570 Bremerhaven
Telephone: +49(471)4831-1845

Fax: +49(471)4831-1271

Model setup and spin-up

For the evaluation of the relationship between enhanced sea-ice export through Fram Strait and enhanced blocking situation, the Finite-Element Sea-Ice Ocean Model (FESOM) is used with a setup configuration of increased resolution in the Northern Hemisphere deep-water formation areas[1, 2]. FESOM is developed at the Alfred Wegener Institute for Polar and Marine Research and uses an unstructured triangular surface mesh approach, which allows to faithfully resolve coastlines and areas of interest in an otherwise coarser global configuration[3, 4, 5]. The model setup has a resolution of around 20-50 km along the coasts of the Arctic and ~ 80-100 km in the interior of the Arctic Ocean (Figure S1) and is forced with the data of the Comprehensive Ocean Ice Reference Experiment version 2 (COREv2) for the 1958-2009 period. The raw atmospheric state in COREv2 data combines NCEP reanalysis[6] with satellite data, with the details of the combination motivated by certain limitations of the reanalysis.

The FESOM setup used in this study is described in more detail in[1, 2]. It was evaluated successfully regarding a reliable sea-ice distribution, general ocean circulation and deep water formation in the Labrador Sea. Figure S3 presents the simulated (a) and observed[7, 8] (b) mean sea-ice concentration fields for the Arctic averaged for the years 1979-2000, indicating an overall good agreement. To bring the model into a quasi-equilibrium state we choose as spin-up strategy to split the process into several spin-up cycles. While in the first cycle the model is initialized with the hydrography of the WOA 2001[9] and subsequently driven by the COREv2[10] data for the period 1958-2009, the second spin-up cycle is initialized with the last output year of the previous cycle and so on. We applied five spin-up cycles over the period 1958-2009, which corresponds to 260 simulated model years and was sufficient to produce an ocean state that is well comparable to observational data[1, 2].

References

1. Scholz, P., Lohmann G., Wang Q. *&* Danilov S. Evaluation of a Finite-Element Sea-Ice Ocean Model (FESOM) setup to study the interannual to decadal variability in the deep-water formation rates, Ocean Dyn., 63*(*4*),* 347–370*, doi:*10.1007/s10236-012-0590-0 *(*2013*)*

2. Scholz, P., Kieke D., Lohmann G., Ionita M. *&* Rhein M. Evaluation of Labrador Sea Water formation in a global Finite-Element Sea-Ice Ocean Model setup, based on a comparison with observational data, J. Geophys. Res. Oceans, 119, 1644–1667, *doi:*10.1002/2013JC009232. *(*2014*)*

3. Danilov S, Kivman G, *and* Schröter J. A finite element ocean model: principles and evaluation. Ocean Model 6:125–150 (2004)

4. Timmermann, R., Danilov S., Schröter J., Böning C., Sidorenko D. & Rollenhagen K. Ocean circulation and sea ice distribution in a finite element global sea ice-ocean model, Ocean Modell., 27*(*3–4*),* 114–129*,* (2009)

5. Wang Q., Sidorenko D., Danilov S., Schroter J. & Jung T. Long term ocean simulation in FESOM: evaluation and application in studying the impact of Greenland Ice Sheet melting. Ocean Dyn 62:1471–1486. doi: 10.1007/s10236-012-0572-2 (2012)

6**.** 53. Kalnay, E., et al. The NCEP/NCAR 40-Year Reanalysis Project, Bull. Amer. Meteor. Soc*., 77*, 437471 (1996)

7. Cavalieri, D., Parkinson C., Gloersen P. & Zwally H.J. Sea ice concentration from Nimbus-7 SMMR and DMSP SSM/I-SSMIS passive microwave data, January 1979–June 2006. National Snow and Data Center, Digital Media, Boulder, CO, USA (2007)

8. Meier, W., Fetterer F., Knowles K., Savoie M. & Brodzik M.J. Sea ice concentration from Nimbus-7 SMMR and DMSP SSM/I passive microwave data, July–December 2006. National Snow and Data Center, Digital Media, Boulder, CO, USA (2007)

9. Conkright, M., Locarnini, E.R., Garcia H., O'Brien T., Boyer T. P., Stephens C. & Antonov J. World Ocean Atlas 2001: Objective Analyses, Data Statistics and Figures. National Oceanographic Data Center, Silver Spring, MD, CD-ROM Data Set Doc., 17 pp. (2002)

10. Griffies, S. M., Winton M., Samuels B., Danabasoglu G., Yeager S., Marsland S., Drange H. & Bentsen M. Datasets and protocol for the CLIVAR WGOMD Coordinated Ocean-sea ice Reference Experiments (COREs), WCRP Report No. 21/2012, pp. 21 (2012)

11. Schmith, T. & Hansen C. Fram Strait ice export during the nineteenth and twentieth centuries reconstructed from a multiyear sea ice index from Southwestern Greenland, Journal of Climate, Vol.16, pp. 2782-2792 (2003)

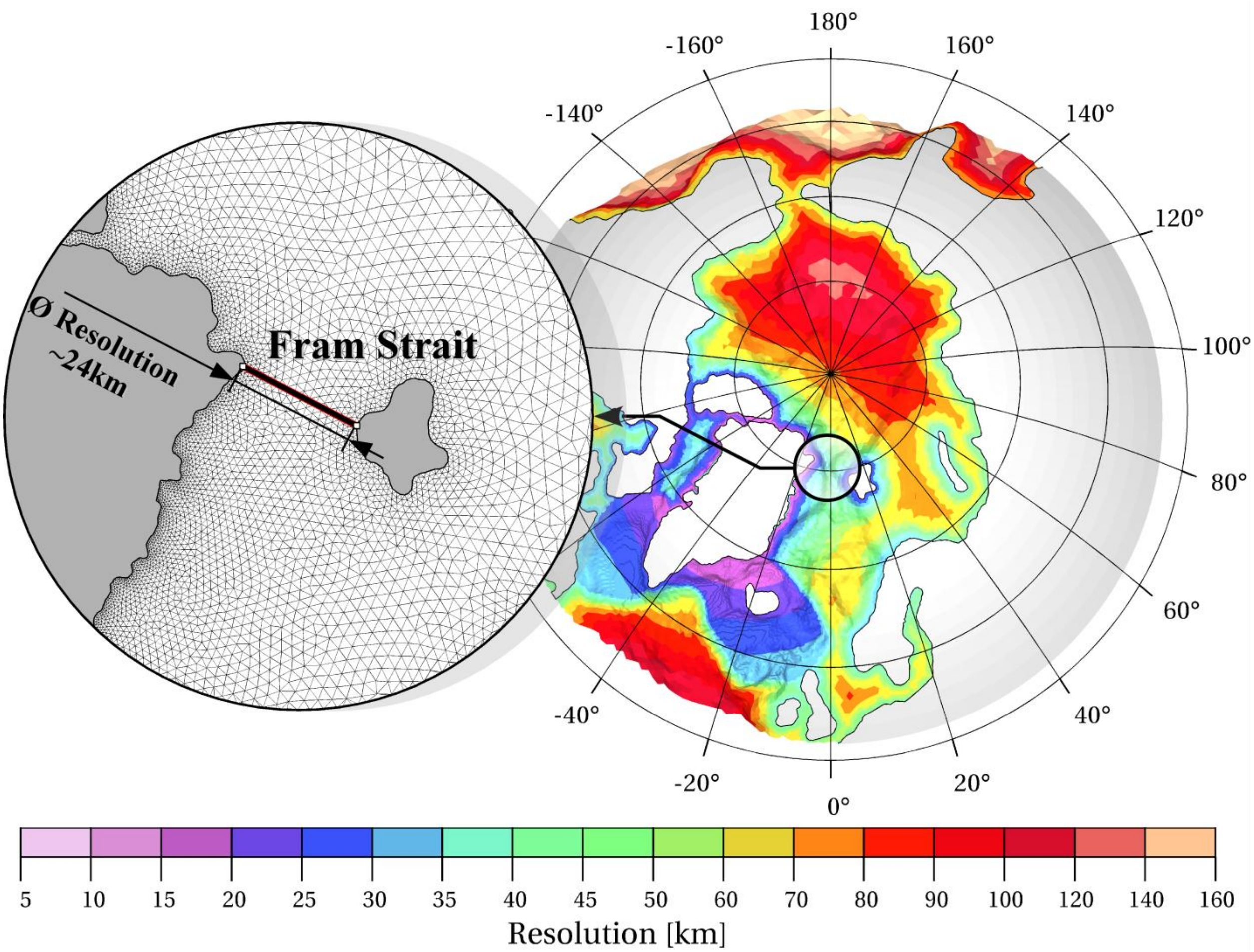

Figure S1│ Northern hemispheric high latitude resolution of the model set-up with increased resolution in Labrador Sea, in Greenland Sea as well as in the coastal areas. Zoomed in is the location of the Fram Strait cross-section with the underlying local mesh. The mean resolution of the model setup within the Fram Strait cross-section is ~24 km[1].

Figure S1 has been produced with MATLAB software – version 2014b (http://de.mathworks.com/products/new_products/release2014b.html)

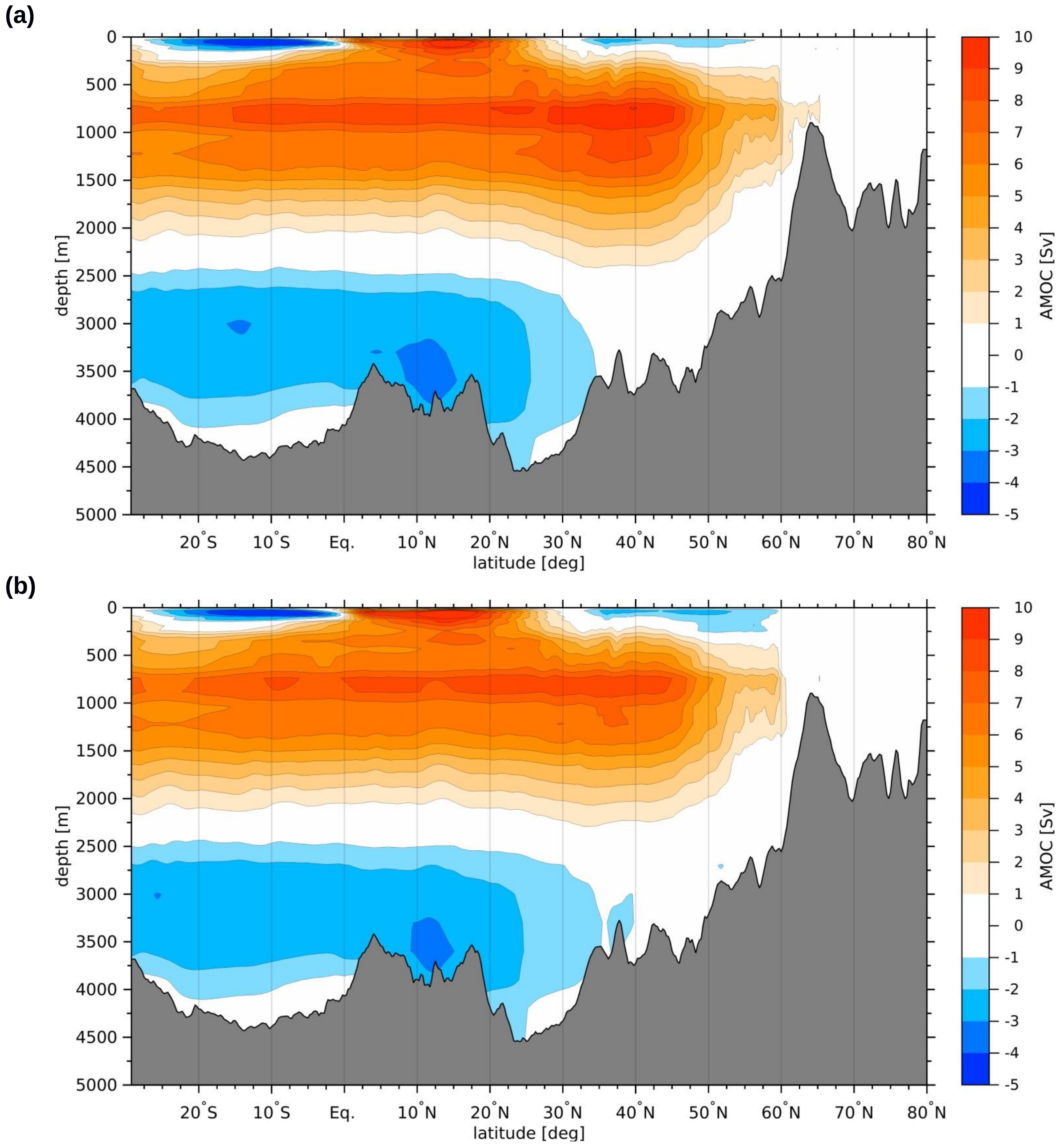

Figure S2│ The mean AMOC streamfunction over the periods: a) 1962 – 1970 and b) 1972 – 1986.

Figure S2 has been produced with MATLAB software – version 2014b (http://de.mathworks.com/products/new_products/release2014b.html)

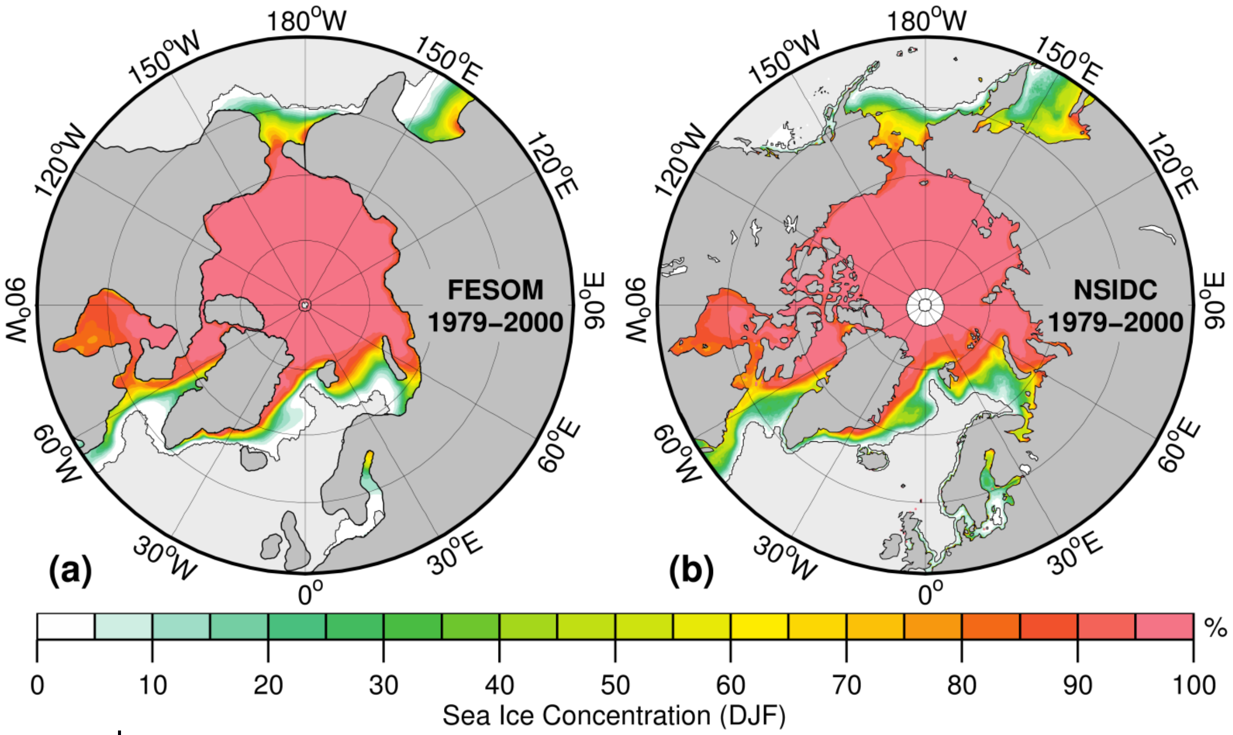

Figure S3│ Simulated (a) and observed (b) mean winter (DJF) sea ice concentration averaged over the period 1979-2000. The observed sea-ice concentration fields have been derived from[7, 8].

Figure S3 has been produced with MATLAB software – version 2014b (http://de.mathworks.com/products/new_products/release2014b.html)

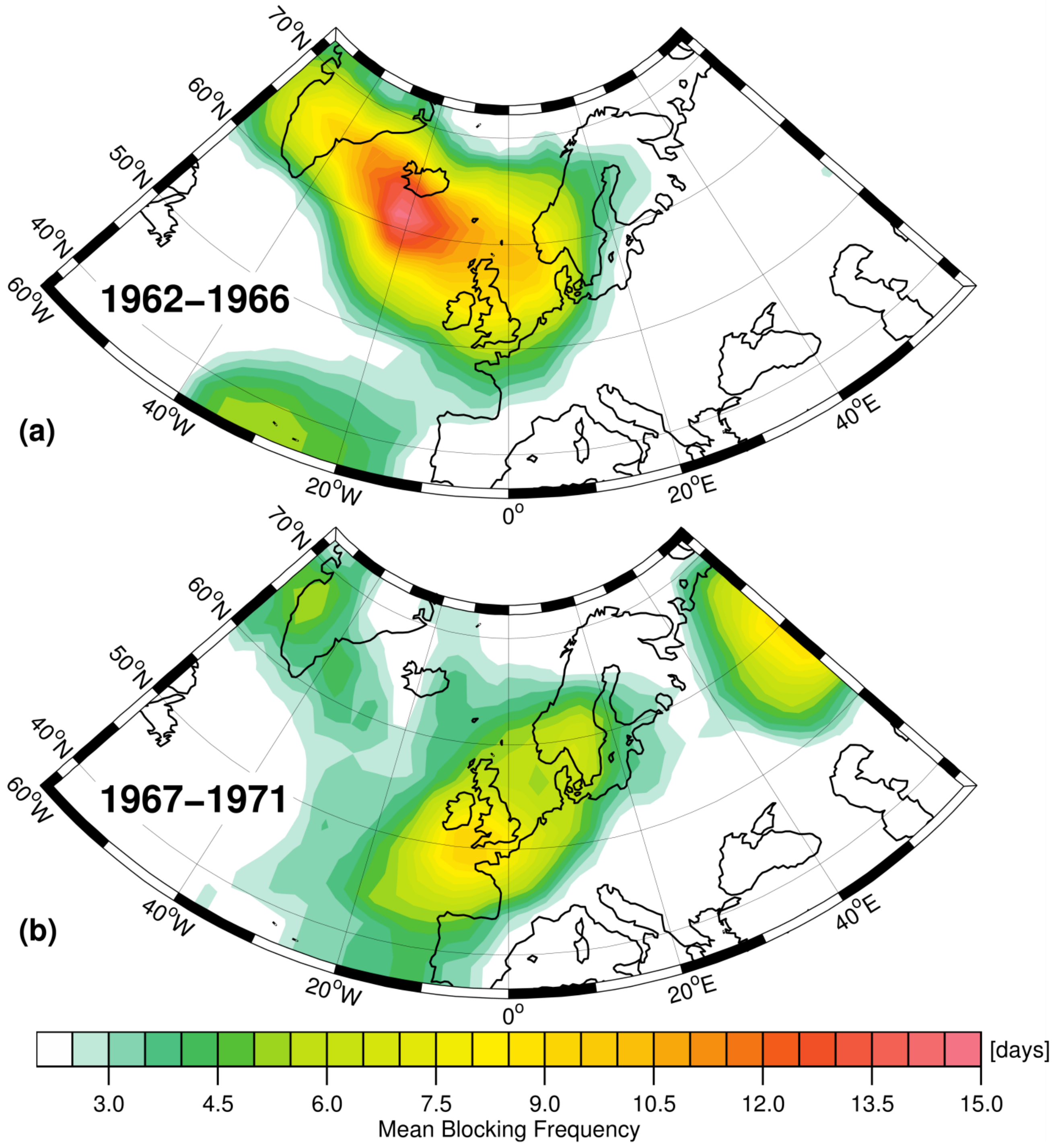

Figure S4│ (a) Winter mean (DJF) 2D atmospheric blocking frequency for the 5 years interval of low sea ice export through Fram Strait (1962-1966) and (b) during the 5 years interval of high Fram Strait sea ice export (1967-1971).

Figure S4 has been produced with MATLAB software – version 2014b (http://de.mathworks.com/products/new_products/release2014b.html)

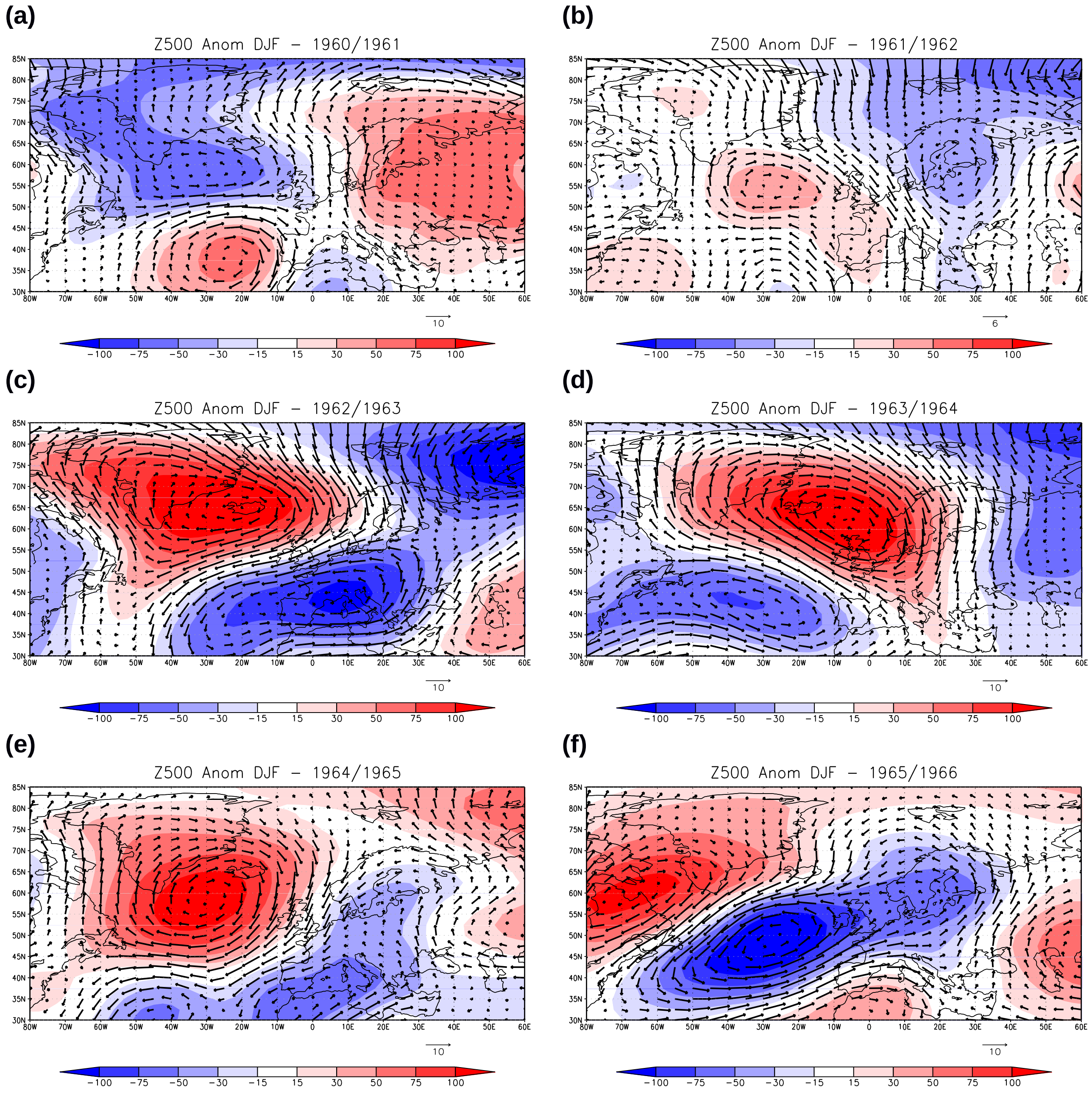

Figure S5│ 500-hPa geopotential height (gpm; shaded) and wind vectors (m/s) winter (DJF) anomalies from the normal 1961–90 for the (a) winter 1960/61; (b) winter 1961/62; (c) winter 1962/63; (d) winter 1963/64; (e) winter 1964/65 and (f) winter 1965/66. Data obtained from The NCEP/NCAR Reanalysis Project.

Figure S5 has been produced with MATLAB software – version 2014b (http://de.mathworks.com/products/new_products/release2014b.html)

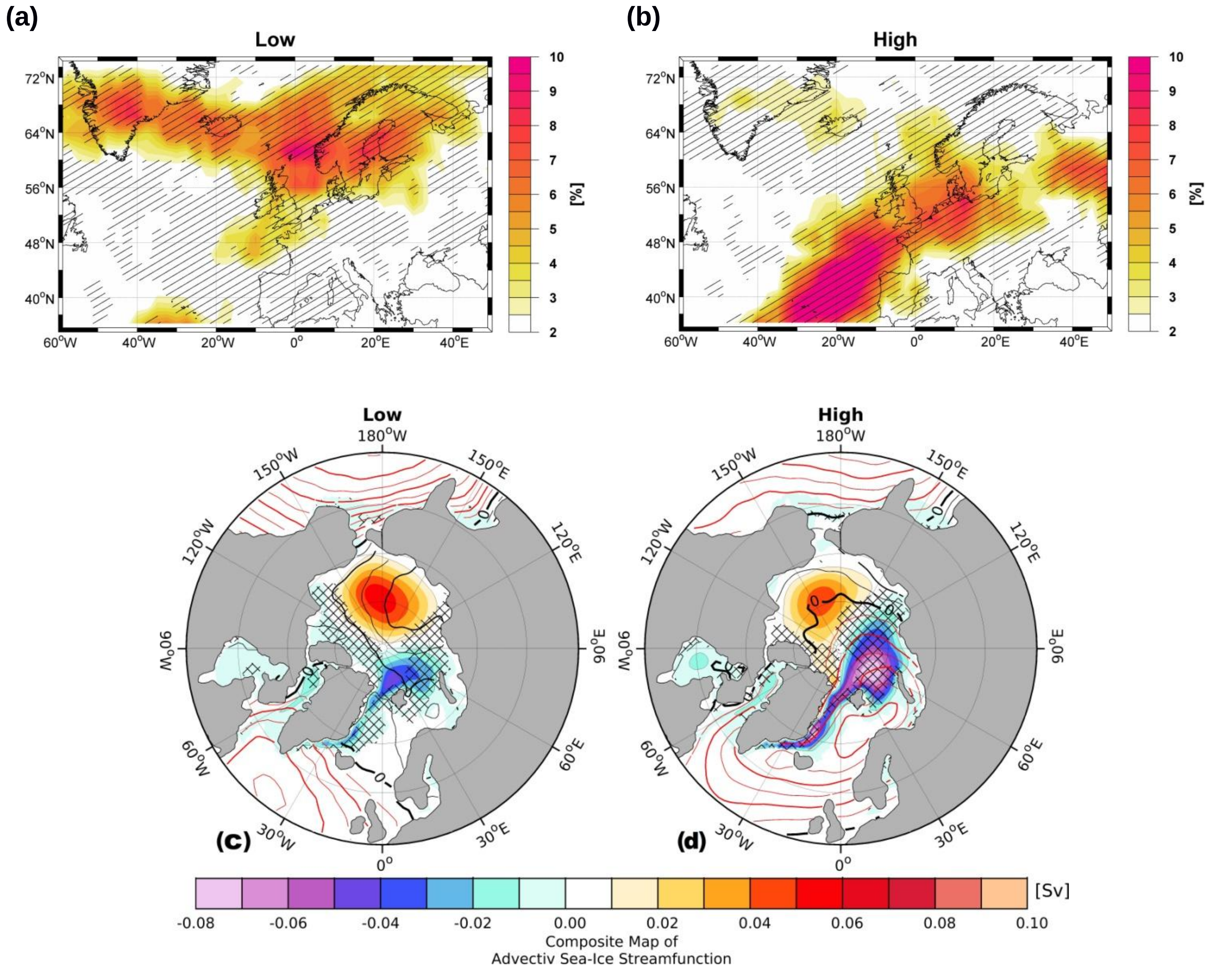

Figure S6│ (a), (b): Atmospheric blocking frequency winter (DJF) simulated composite maps with respect to the modeled DJF Fram Strait sea-ice export time-series below (Low < -0.75 standard deviation, (a)) and above (High > 0.75 standard deviation (b)) ; (c), (d) same as (a), (b) but for the advective sea ice streamfunction. Contour lines mark the high composite map of the SLP anomaly (units: hPa), where black lines indicate positive and red lines negative anomalies with an interval of 1.5 hPa. The hatching highlights significant anomalies at a confidence level of 95%.

Figure S6 has been produced with MATLAB software – version 2014b (http://de.mathworks.com/products/new_products/release2014b.html)

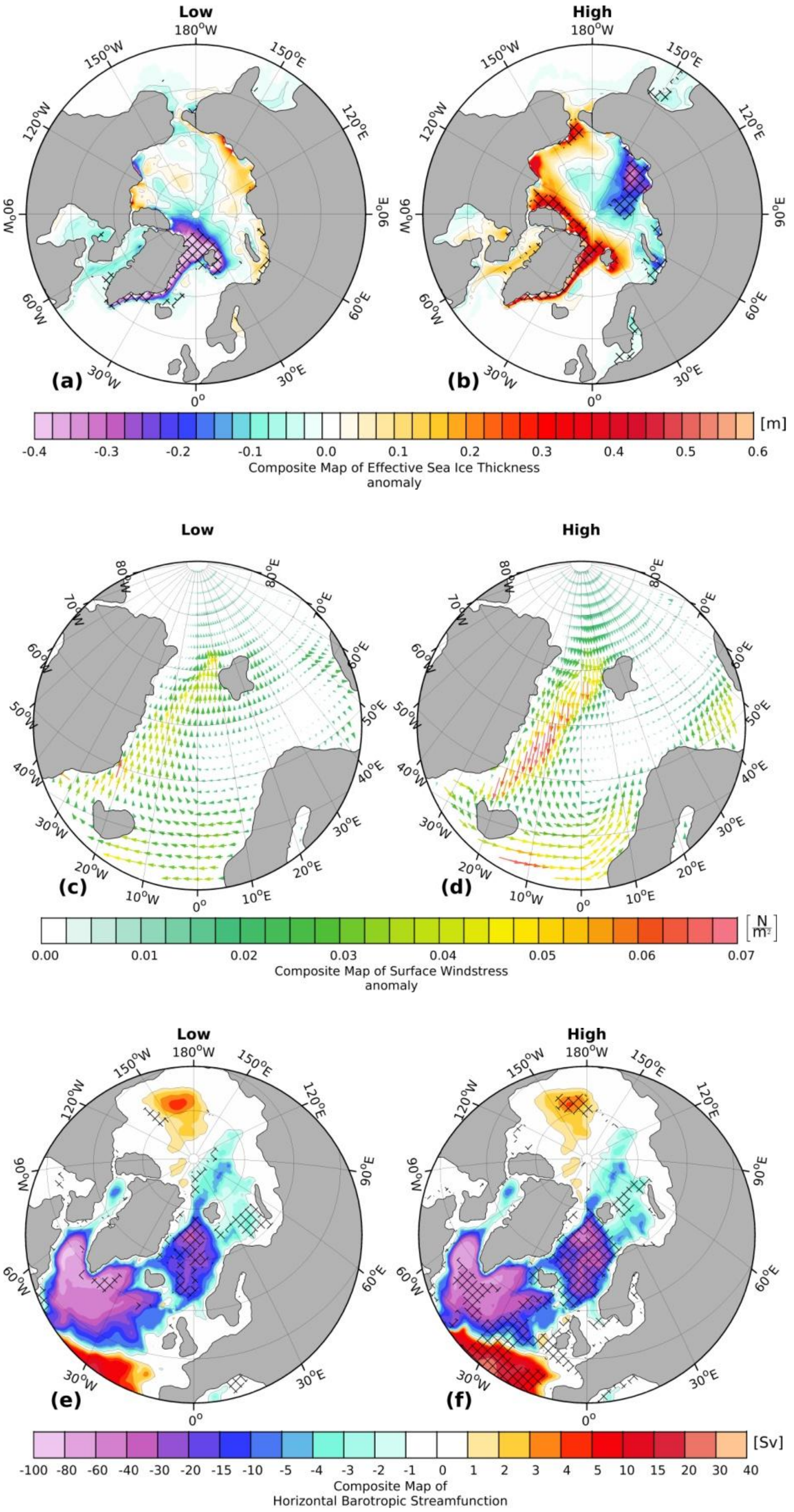

Figure S7│ (a), (b): Sea-ice thickness simulated winter (DJF) anomaly composite maps with respect to the modeled DJF Fram Strait sea-ice export time-series below/above 0.75 standard deviation (a) Low composite and (b) High composite; (c), (d) same as (a), (b) but for the surface wind-stress anomaly; (e), (f): same as (a), (b) but for the horizontal barotropic ocean streamfunction . The hatching in (a)-(f) highlights significant values at a confidence level of 95%.

Figure S7 has been produced with MATLAB software – version 2014b
(http://de.mathworks.com/products/new_products/release2014b.html)

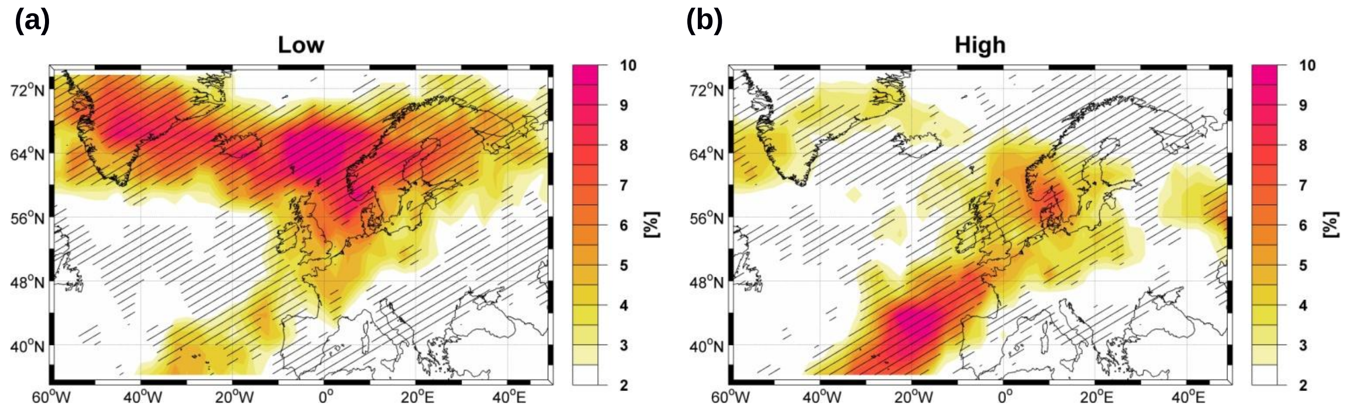

Figure S8│ 2D Atmospheric blocking frequency composite maps for winter (DJF), with respect to the reconstructed DJF Fram Strait[11] sea-ice export: (a) Low Composite (FSSIE < -0.75 standard deviation) and (b) High Composite (FSSIE > 0.75 standard deviation). The hatching in (a)-(b) highlights significant anomalies at a confidence level of 95%.

Figure S8 has been produced with MATLAB software – version 2014b (http://de.mathworks.com/products/new_products/release2014b.html)

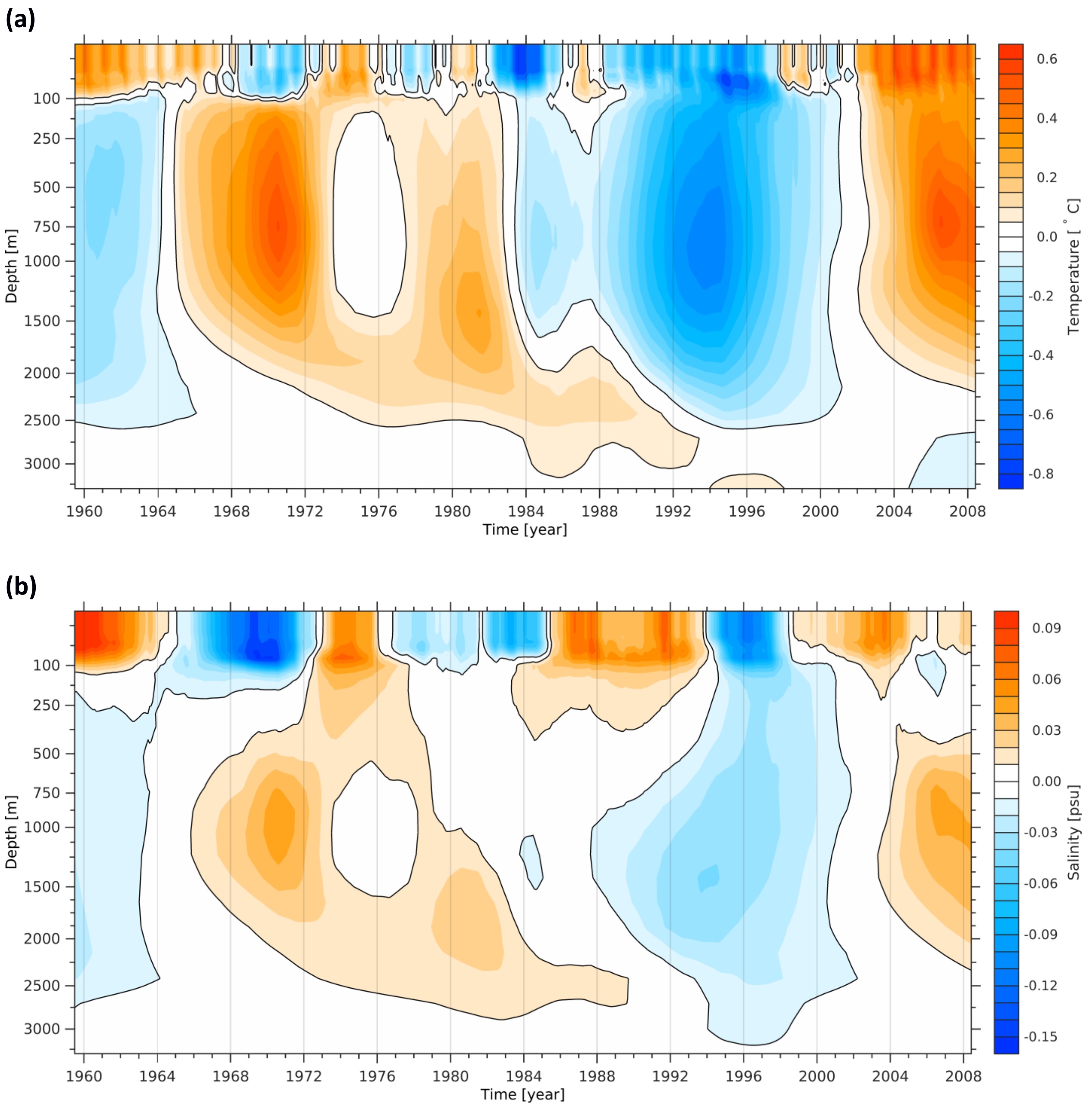

Figure S9│ The modeled anomalous temperature (a) and salinity (b) over depth and time averaged over the central Labrador Sea. The data are detrended and smoothed with a 3-year-running-mean filter.

Figure S9 has been produced with MATLAB software – version 2014b
(http://de.mathworks.com/products/new_products/release2014b.html)

Table S1. Correlation coefficients between the observed FSSIE (Schimth and Hansen, 2003) and the seasonal modeled FSSIE.

	DJF	MAM	JJA	SON	Annual
r	0.61^{***}	0.15	0.37^{*}	0.07	0.47^{**}

where r = correlation coefficient, DJF – winter (December – January – Feburary) FSSIE mean, MAM – spring (March – April – May) FSSIE mean, JJA – summer (June – July – August) FSSIE mean and SON – autumn (September – October – November) FSSIE mean.
*** = 0.001 significance level (99.9%), ** = 0.01 significance level (99%) and * = 0.05 significance level (95%).
